# Measuring attitudes towards interprofessional learning. Testing two German versions of the tool "Readiness for Interprofessional Learning Scale" on interprofessional students of health and nursing sciences and of human medicine 

**DOI:** 10.3205/zma001110

**Published:** 2017-08-15

**Authors:** Christiane Luderer, Matthias Donat, Ute Baum, Angelika Kirsten, Patrick Jahn, Dietrich Stoevesandt

**Affiliations:** 1 Martin-Luther-University Halle-Wittenberg, Faculty of Medicine, Institute for Health and Nursing Science, Halle (Saale), Germany; 2 Martin-Luther-University Halle-Wittenberg, Faculty of Philosophy – Educational Sciences, Institute for Pedagogy, Halle (Saale); Germany; 3 University Clinic Halle A.ö.R., Head of Administration Healthcare Research, Halle (Saale), Germany; 4 Martin-Luther-University Halle-Wittenberg, Faculty of Medicine, Head of Dorothea-Erxleben-Learning-Center, Halle (Saale), Germany

**Keywords:** interprofessional learning, testing of instruments, attitude measurement, health professions

## Abstract

**Objective:** In order to verify the methodological quality of two versions of a tool for measuring attitudes towards interprofessional learning, we adapted – in terms of translation and scale form – the Heidelberg Version [1] of *Readiness for Interprofessional Learning Scale* - RIPLS [2], a methodologically controversial tool that had been translated into German, and compared both the original and new versions.

**Method: **Three items were reworded and the scale form altered (from five to four levels), leading to the Halle Version that was validated by means of a cognitive pretest (*n*=6). Both questionnaires were completed by students taking the interprofessional degree program in Health and Nursing Sciences (HNS) and by students of Human Medicine. The test quality of both tools was examined by analyzing the main components and reliability using the scales allocation of the items as according to Parsell and Bligh [2].

**Results: **The questionnaires were randomly assembled and distributed to 331 students. The response was *n*=320 (HNS *n*=109; Medicine *n*=211). The Halle Version “RIPLS-HAL” of the questionnaire was completed by *n*=166 and the Heidelberg Version “RIPLS-HDB” by *n*=154. In the main component analysis the data could not depict the scale patterns of the original Australian tool. The reliability values of both the Heidelberg and Halle versions were only satisfactory for the “Teamwork and Collaboration” and “Professional Identity” scales.

**Conclusions: **The German version of the Readiness for Interprofessional Learning Scale has only limited suitability for recording the attitude towards interprofessional learning. The present versions can be regarded as an approach towards developing a more suitable tool.

## Zusammenfassung

**Ziel: **Um die methodische Qualität eines Instrumentes zur Messung der Einstellung zum interprofessionellen Lernen in zwei Versionen zu überprüfen, wurde die Heidelberger Version [2] der ins Deutsche übersetzten und methodisch umstrittenen *Readiness for Interprofessional Learning Scale* - RIPLS [3] in Bezug auf Übersetzung und Skalenform angepasst und beide Versionen wurden verglichen.

**Methode: **Umformulierungen in drei Items und die Veränderung des Skalenformats (von fünf- zu vierstufig) führten zur Hallenser Version, die mittels kognitivem Pretest (*n*=6) validiert wurde. Die beiden Fragebögen wurden von Studierenden des interprofessionellen Studiengangs Gesundheits- und Pflegewissenschaften (GPW) und von Studierenden der Humanmedizin ausgefüllt. Die Testqualität der Instrumente wurde mittels Hauptkomponenten- und Reliabilitätsanalysen anhand der Skalenzuweisung der Items nach Parsell und Bligh [2] untersucht.

**Ergebnisse: **Die Fragebögen wurden randomisiert gestapelt und an 331 Studierende ausgegeben. Der Rücklauf lag bei *n*=320 (GPW *n*=109; Medizin *n*=211). Davon füllten *n*=166 den Bogen der Hallenser Version „RIPLS-HAL“ und *n*=154 den Bogen der Heidelberger Version „RIPLS-HDB“ aus. Die Daten konnten in Hauptkomponentenanalysen die Skalenbildung des australischen Originals nicht abbilden. Die Reliabilitätswerte sowohl der Heidelberger als auch Hallenser Version sind nur für die Skalen „Teamwork and Collaboration“ und „Professional Identity“ akzeptabel.

**Schlussfolgerungen: **Die Readiness for Interprofessional Learning Scale in der deutschen Version ist aufgrund methodischer Limitationen bedingt für die Erfassung der Einstellung zum interprofessionellen Lernen geeignet. Die derzeitigen Versionen können als Ansatz zur Entwicklung eines geeigneteren Instrumentes betrachtet werden.

## Background

In medicine, interprofessional learning is defined as “learning from, with and about each other through interacting between members of two or more professions – either as a product of vocational training or as a spontaneous happening” [3]. Interprofessional learning is recommended in order to improve cooperation and quality in healthcare [4]. The varying structures of the qualifications in the health profession in Germany make continuous shared learning over a longer period difficult [5], so that up to now there are only insufficient data available regarding the willingness or readiness in the individual healthcare professions to take part in interprofessional learning. This willingness can result or be brought about, for example through measuring the attitudes towards interprofessional learning.

Attitudes are to be understood as multi-dimensional, hard to operationalize systems that influence actions and behavior indirectly and interpersonally in a variety of ways [6]. It is difficult to demonstrate a direct interdependence between knowledge, attitude and behavior and this is also not inevitably found in clinical decision-making [7]. Nevertheless, attitudes and stereotypes with respect to colleagues in one’s own profession or in other professional groups are looked on as important factors for the success of teamwork [8]. The attitude towards interprofessional learning represents a prerequisite that promotes learning achievement and the development of one’s own professional identity as opposed to the other professions in the team [9], [10].

Since 1999, the Martin-Luther-University Halle-Wittenberg has offered an interprofessional study course in Health and Nursing Sciences (HNS). Students in nursing, midwifery and therapy professions learn together in the Faculty of Medicine and carry out clinical and scientific projects together. Compliant with the position paper on interprofessional education in healthcare professions [11], course units for HNS and medicine students were conceptualized in 2013 and have been provided since then by an interprofessional workgroup. Since 2016, within the frame of a project supported by the Robert Bosch Foundation, new learning stages are being developed and provided as compulsory teaching programs. Scientific monitoring is being used to register the impact of interprofessional teaching on the attitudes of students towards interprofessional learning during their studies and beyond. In order to depict the attitudes to interprofessional learning, a tool is required that on the one hand does justice to the complexity of measuring attitudes and has on the other hand good practicability, so that it can be used regularly in the evaluation of the courses. A test run with appropriate tools served at the same time as an upfront inventory.

The tool for measuring the readiness for interprofessional learning (RIPLS) [2] that was published by Parsell and Bligh in 1999 has already been translated into German; however, tests have not shown a clear recommendation for RIPLS [1]. Nevertheless, due to intensive use internationally and to the predominantly positive experience gained [12], the RIPLS has been applied and modified, as agreed on with the translators of the Heidelberg Version (RIPLS-HDB). The new Halle Version (RIPLS-HAL) differs from the original in that three items have been reworded and the scale form altered. Both tools were tested by a cohort of medical students and in the HNS study courses.

## State of Research

The effects of interprofessional learning are increasingly becoming a popular subject in publications, of which only a few do justice to the character of interprofessional learning as a complex intervention with sustainable effects all the way into clinical practice [13]. Several publications report significant findings with larger samples when using tools such as RIPLS [14], [15]; others have a conceptual character [16] and refer to the effects of specific didactical approaches of interprofessional learning [17] or focus on the students’ satisfaction [18]. In their systematic review, Reeves et al. [19] confirmed the necessity of giving future studies on this theme a distinct profile in order to demonstrate the marked impact of interprofessional learning. 

Tannhauser et al. have searched through various instruments for interprofessional learning and cooperation, recording not only attitudes and perceptions but also factors of interaction [20]. Twenty-three studies were examined, from which six tools dealing with interprofessional learning were identified where RIPLS [2] and IEPS (Interdisciplinary Education Perception Scale) [21] were two tools which had already been sufficiently evaluated [20]. In comparison, a similar methodological quality with a different usage can be seen: while the RIPLS tool covers one’s own willingness towards shared learning, IEPS is more suitable for advanced learners who reflect their own perception of teamwork more consciously [22].

One of the most applied tools for evaluating interprofessional education processes is the scale developed and tested by Parsell and Bligh [2], which is internationally well known [1], [12]. This is followed by several translations with heterogeneous statements about the test quality: the Australian original was tested as satisfactory, whereby the three-factorial variant seemed doubtful [23]. A positive evaluation was given to the Japanese translation [24] and to a Canadian French version [25], but the factor structure of the original version could not be confirmed for these. The present German version RIPLS-HDB by Mahler et al. (2014) is seen as methodically critical [1] and the same applies to the Swedish version [26]. Whereas the RIPLS authors characterize the tool as a readiness tool, most of the items refer back to attitudes towards interprofessional learning from which readiness can be derived. For this reason, RIPLS in this project has been characterized and applied as an attitude tool. The study’s aim was to determine whether the adapted RIPLS-HAL (adapted in translation and scale form) leads to a better measurement quality in comparison to RIPLS-HDB.

## Methodical approach

### Instruments applied for measuring the attitude towards interprofessional learning

The Australian Version of RIPLS [2]

The Australian scale is intended for registering the readiness for interprofessional learning. It consists of the following three subscales [2]: 

“Teamwork and Collaboration”: The nine items in this subscale represent the assumption that shared learning has advantages. Six of the items focus on acquiring team competences and using these for specific goals. The three other items pursue the construction of relationships with prospective physicians and other healthcare professionals. All of the items express positive aspects of teamwork and collaboration.

“Professional Identity”: This subscale with its three negative and four positive items intends capturing statements concerning professional identity. The items refer to task areas of learning and working and to the assessment of the advantages of interprofessional learning. Whereas the negatively expressed items are oriented towards waste of time and the non-necessity of interprofessional learning or mono-professionalism, some of the advocating items are, among others, communicative and interprofessional competence as well as the positive appreciation of interprofessional group work. 

„Roles and Responsibilities“: The shortest of the three subscales consists of only three items, which target professional self-conception and confidence in the roles of one’s own profession in healthcare. Thus, the scale is devoted to the contradictions resulting from shared learning and the partly contrasting situations found in clinical practice.

All of the RIPLS subscales have a five-step response format (“Strongly agree”, “Agree”, “Undecided”, “Disagree”, “Strongly disagree”). 

The German translation of RIPLS [1]

The RIPLS developed by Parsell and Bligh [2] was first translated into German by the Department of General Medicine and Healthcare Research at the University Clinic Heidelberg [1]. Translated in a multi-stage process, the scale compiles the same 19 items as the original. The three subscales and the five-step response format (“Strongly agree”, “Agree”, “Undecided”, “Disagree”, “Strongly disagree”) of the original version were also adopted.

#### Adaption of the German version of RIPLS 

The adaption of the Heidelberg Version to create the Halle Version was performed by an interprofessional group of experts from the University Halle-Wittenberg (*n*=7) that included colleagues from the Departments of Medicine, Nursing Science, Teaching Methodology in Health Professions, Pedagogical and Clinical Psychology as well as from the research field of decision-making and attitude measurement. Particular attention was paid to the content validity of the tool. The content validity of RIPLS-HAL at item level could on the whole be proven. The identification of RIPLS-HAL as a tool for registering the readiness, i.e. preparedness or intention towards a certain behavioral pattern was discussed and because of the distinct focus on the positive or negative evaluation of interprofessional learning and its impact, the RIPLS was characterized mainly as an instrument for measuring attitudes.

The adaption consisted of changing the response scale to a four-level format, similar to dis-/agree in the original (“Disagree”, “Somewhat disagree”, “Somewhat agree”, “Agree”), in order to take the decision-making impact of an even number of responses into consideration [27]. In addition, the language of some of the items was modified, which can be seen in Table 1 (Tab. 1). The aim here was to ensure that the contents of the items were understood. For example, the expression “health problems” would be understood as a professional instruction by members of the healthcare professions in Germany (cf. ICD, [28], whereas “patients’ problems” would be more likely be perceived as a task for social care.

Since uneven scale formats are not undisputed [29] and the methodological effects of changing the scale format with regard to attitude measurement are still under discussion [30], it was important to find out to what extent response behavior would change if the scale format was altered from an uneven number to an even number of responses. After consulting the Department for General Medicine and Healthcare Research at the University Clinic Heidelberg, it was decided to test both versions. The objective was on the one hand to examine the impact of the change in the response format on the response behavior and on the validity of the tool. On the other hand, the German version by Mahler et al. [1] was checked solely by non-medical healthcare professions. This was the first time in which a survey of this size was carried out comparatively with medical students and students of other healthcare professions in one faculty.

#### Cognitive pretest of RIPLS-HAL

Using the “Think aloud” method, a cognitive pretest was performed on the modified tool to check the validity, applicability and linguistic comprehensibility [31]. This qualitative cognitive procedure enables insight into the reflective processes during answering. The cognitive pretest took place during a voluntary random survey and included professionally experienced students in the Master Course for Health and Nursing Science at the Martin-Luther-University Halle-Wittenberg (two physiotherapists, three nurses and a midwife; *n*=6). Apart from requesting the participants to make their thoughts transparent regarding the purpose and comprehensibility of the questions, they were also asked about the purpose and reasoning behind their responses. Since the cognitive pretest with all the respondents confirmed the comprehensibility and ease of handling the questionnaire, the tool was released for standardized testing. 

#### Sample and access to the field

A random sample was recruited from the Medical Faculty of the Martin-Luther-University Halle-Wittenberg and consisted of medical students and interprofessional HNS students who were given the questionnaires during specific course lectures that took place in October and November 2014.

#### Data transmission and quality check

The questionnaires were created using interview software and transferred to a randomized stack (randomization by means of an electronically generated randomized list). The completed questionnaires were then scanned in, opposing items were reversed and integrated in the list of categories and finally a random check for transmission errors was performed which showed no quality defects.

#### Data analysis

The data analysis was aimed at determining the methodological quality of both RIPLS versions. To check the factor structure, an explorative analysis of the main components was carried out with a factor extraction method, in which the correlation matrix of the data was checked by means of the Kaiser-Meyer-Olkin criterion and the suitability of the sample determined by means of the Bartlett Test [32], [33], [34]. The reliability was defined by the internal consistencies (Cronbachs Alpha) [35]. The IBM Statistics SPSS 22 software was used for the data analysis.

## Results

### Response and description of the sample

A total of 331 questionnaires were distributed (medicine: 220; HNS: 111) and completed with a response of 96% (*n*=320). The responses consisted of 166 questionnaires in version RIPLS-HAL and 154 in version RIPLS-HDB.

The basic data set contained socio-demographic data such as age, gender, current year of study or training, and profession before starting to study. Compared with the HNS students (*n*=109), the medical students (*n*=211) in both groups were represented nearly twice as often. This reflects the actual distribution of the students in the Faculty of Medicine. Table 2 (Tab. 2) shows the descriptive characteristics of both samples.

#### Factor-analytical view of the RIPLS Items – RIPLS-HDB

To check the structure of the original RIPLS items [2] in the translation by Mahler et al. [1], an principal components analysis was carried out. Factor analysis for RIPLS-HDB have not been performed up to now; this is a first report. Since the factor structure of a questionnaire following a translation cannot inevitably be reproduced due to cultural and linguistic differences, an exploratory factor analysis was carried out instead of following a confirmatory procedure. The evaluation strategy used by Parsell and Bligh [2] was selected since this enabled a comparison of the results. This strategy consists of an analysis of the main components followed by Varimax rotation. Missing item responses were replaced by the respective item mean value. 

The Kaiser-Meyer-Olkin Index (=.76) resulted in an average sample suitability; the Bartletts’ Test (*p*<.001) proved that the items were suitable for performing a main component analysis [33]. Seven factors with eigenvalues larger than one resulted from the initial main component analysis: λ_1_=4.23; λ_2_=2.05; λ_3_=1.41; λ_4_=1.20; λ_5_=1.12; λ_6_=1.05; λ_7_=1.003. The parallel analysis according to Horn [34] indicated two factors, since the empirically determined eigenvalue of the third factor was barely under the corresponding randomly determined eigenvalue (=1.44). Determining the number of factors by means of the eigenvalue progression (Scree Analysis, [36]) did not produce a definite result. Due to the original construction idea of the tool by Parsell and Bligh [2] consisting of three subscales, three factors were still assumed in the further calculations.

In the subsequent main component analysis with Varimax rotation the three factor solution (see Table 3 (Tab. 3)) resulted in an explained variation of 40%. It was seen that with one exception (TC8) the items of the subscale “Teamwork and Collaboration” showed the highest loadings on the first factor each time. Three of these items (TC2, TC7, TC9) showed only loadings of *l*<.50 on this factor. As expected, the second factor was substantially fed only by items PI2 and PI3 from the subscale “Professional Identity”, whereas the highest loading was shown on the third factor by item PI1 and on the first factor by the remaining items of the subscale, respectively. The items of the subscale “Roles and Responsibilities” had the highest loadings on the third factor, as expected, whereby only the loading of item RR3 was larger than .50.

In the reliability analysis using the items scales acc. to Parsell and Bligh [2] and following re-coding of negatively formulated items, the following statistical values resulted (Cronbachs Alpha): for Teamwork and Collaboration: α=.71; for Professional Identity: α=.61; for Roles and Responsibilities: α=-.27 (without re-coding RR2: α=-.38).

#### Factor-analytical view of the RIPLS Items – RIPLS-HAL

An exploratory analysis of the main components was also carried out to check the structure of the modified RIPLS version. Missing item responses were replaced by the respective item mean value. The Kaiser-Meyer-Olkin Index (=.78) also showed an average sample suitability and the Bartletts’ Test (*p*<.001) proved that the items were suitable for performing a main component analysis [33]. Six factors with eigenvalues larger than one resulted from the initial main component analysis: λ_1_=4.27; λ_2_=1.68; λ_3_=1.64; λ_4_=1.15; λ_5_=1.08; λ_6_=1.01. The parallel analysis according to Horn [34] indicated three factors for RIPLS-HAL, since the empirically determined eigenvalue of the third factor laid under the corresponding randomly determined eigenvalue (=1.34). Determining the number of factors by means of the eigenvalue progression (Scree Analysis, [36]), however, pointed rather to one factor. Returning to the original construction idea of the tool by Parsell and Bligh [2] with three subscales, three factors were still assumed in the further calculations here, too.

In the subsequent main component analysis with Varimax rotation, the three factor solution (see Table 4 (Tab. 4)) resulted in an explained variation of 40%. It was seen that five items of the subscale “Teamwork and Collaboration” showed the highest loading on the first factor each time, while the items TC4 and TC7 showed loadings of *l*<.50 on this factor. The remaining four items substantially fed an individual second factor. None of the items of the subscale “Professional Identity” showed substantial loadings on the second factor. However, with the exception of item PI3 (highest loading on the third factor), these items loaded on to the first factor. The items of subscale “Roles and Responsibilities” depicted the scale with loadings *l*>.50 on the third factor.

In the reliability analysis using the items scales acc. to Parsell and Bligh [2] and following re-coding of negatively formulated items, the following statistical values resulted (Cronsbachs Alpha): for Teamwork and Collaboration: α=.71; for Professional Identity: α=.68; for Roles and Responsibilities: α=-.09 (without re-coding RR2: α=-.47).

#### Summary of the results

The results show that the factor structures of both tools cannot be depicted with regard to the first and second scales (Teamwork and Collaboration; Professional Identity), although the Heidelberg Version was more clearly structured with regard to the Teamwork and Collaboration scale than the Halle Version in this survey. The third scale (Roles and Responsibilities) was confirmed in both RIPLS versions by means of the loadings in their structure but the low internal consistence (due to the heterogeneous content of the three items) pointed to this scale’s lack of quality. The scales Teamwork and Collaboration as well as Professional Identity indicate a moderate internal consistence with both tools.

## Discussion of the results

The results justify a critical inspection of the German versions of RIPLS with regard to their suitability, as was confirmed by the Heidelberg workgroup who had done the translation [37]. When comparing the main component analysis of both versions (RIPLS-HDB vs. RIPLS-HAL), it is not entirely clear how such differing loadings can occur in a factor analysis when the items are only slightly different. This confirms the scales’ low replication quotient, which is typical for attitude research when existing tools are subsequently tested [38].

A possible explanation for this could lie in the fact that the changed scale format (even vs. uneven) might have led to a change in the response behavior, particularly regarding the reliability and validity [39]. However, it seems strange that such differing loading patterns are found for similar values on internal consistence, e.g. in the scale Teamwork and Collaboration.

The response format of the Halle version (Likert Scale with four-level response format) was chosen to examine the effects of the tendency towards the center, as used in the Heidelberg version (five-level response format). Choosing a six-level response format, as is currently under methodological discussion, might have been an alternative here [40]. However, this form was chosen because attitude measuring in particular is often done with a four- and five-level response format [38], [41]. There is no actual connection to an improvement or deterioration of the internal consistence when compared to RIPLS-HDB but this should be ignored due to the unconfirmed structure of the subscales.

The testing of the internal consistence of the RIPLS-HDB subscales in comparison with the preliminary examinations in 2014 [1] shows clearly how heterogeneous and therefore unreliable the completion of the questionnaires is. This could be due to the composition of the sample in this test, since it deviated from the first RIPLS-HDB survey [1] to the effect that the majority of the participants were medical students whereas in the preliminary surveys no medical students took part. If the medical students’ attitudes differed from those of the other students, who were already studying interprofessional, then this could have led to an inconsistent response behavior. However, this could also be understood as a further point of criticism of RIPLS because the development of such a tool should have the aim of being suitable for registering the attitudes of all those involved (in health and nursing sciences as well as human medicine) towards interprofessional learning. Alternatively, RIPLS versions could be developed which could be used for specific target groups, thus leading to potentially differing but nevertheless valid and reliable results.

Examining the content validity at the item level during the cognitive pretest showed that, in spite of the non-reproducible structure of the subscales, the individual items provide the interested practitioner with a good chance of getting data on the attitude towards interprofessional learning. The tool itself, however, does not do justice to an instrument for measuring attitude because the allocation of the items to the scales according to Parsell and Bligh [2] seems to be random. The content validity of Parsell and Bligh’s three subscales and the tool as a whole should obviously be queried because it cannot be clearly assumed that the scale measures what it purports to measure [1], [2], [37]. 

Apart from this, it ought to be considered whether Parsell and Bligh’s approach [2] in the theoretical conception of RIPLS should – from a psychological point of view – be challenged. The authors developed a tool for registering readiness, i.e. preparedness or intention with respect to a certain behavior. However, according to Fishbein and Ajzen [[42]: S. 39] this contains “the person’s estimate of the likelihood or perceived probability of performing a given behavior.” If the wording of the RIPLS items is examined under this perspective, then the tool does not measure behavioral readiness or intention but rather an attitude towards interprofessional learning. According to Fishbein and Ajzen [[42]: S. 76], an attitude is “a latent disposition or tendency to respond with some degree of favorableness or unfavorableness to a psychological object […] attitudes are evaluative in nature, ascribing to individuals a position on a unitary evaluative dimension with respect to an object […].” If a bipolar evaluative dimension is assumed, then the construction that is outlined in the RIPLS questions should be recognized as having attitude character; phrasing such as “Shared learning will help me to…” or “Shared learning will contribute to…” make this seem likely. It should therefore be considered whether first of all a clear theoretical conception and differentiation should be carried out (attitude vs. readiness) which could then serve as a basis for a valid and reliable measurement.

## Discussion of the methodical procedure

The methodical advantages of the survey are to be seen in a research situation that is homogeneous for all students, in the random distribution of both questionnaires, in the anonymity of the survey and in the only slight probability that the response behavior is due to social desirability.

The cognitive pretest was carried out without medical students. This is mitigated due to the fact that the revision was performed by an interprofessional workgroup to which also physicians belonged.

Since this was a randomized sample with uniquely developed, interprofessional study conditions at a faculty of medicine in Germany, the results can be applied only restrictedly to other student populations.

## Outlook

The Readiness for Interprofessional Learning Scale in the German versions RIPLS-HDB and RIPLS-HAL cannot be fully recommended for registering the attitude towards interprofessional learning and changes therein, for instance in the longitudinal profile of an interprofessional study course. Nor can a selective use of individual subscales be recommended, since neither the validity nor the reliability in both German versions of the tool could be confirmed convincingly. Nevertheless, in Halle (Saale) basic data are now available for use descriptively at least at item-level.

In Germany interprofessional structures in training and study courses are increasing, during the course of which the development and testing of suitable tools should be driven forward. This can be supported by cooperation across institutional boundaries in order to generate enough sample sizes to provide a reliable tool. There are international alternatives to the psychometric measurement of the attitude to interprofessional learning [37] that could enable a speedy evaluation of the effects of interprofessional learning on the attitude of learners in German-speaking countries by means of methodically good translation processes. 

## Competing interests

The authors declare that they have no competing interests.

## Figures and Tables

**Table 1:**
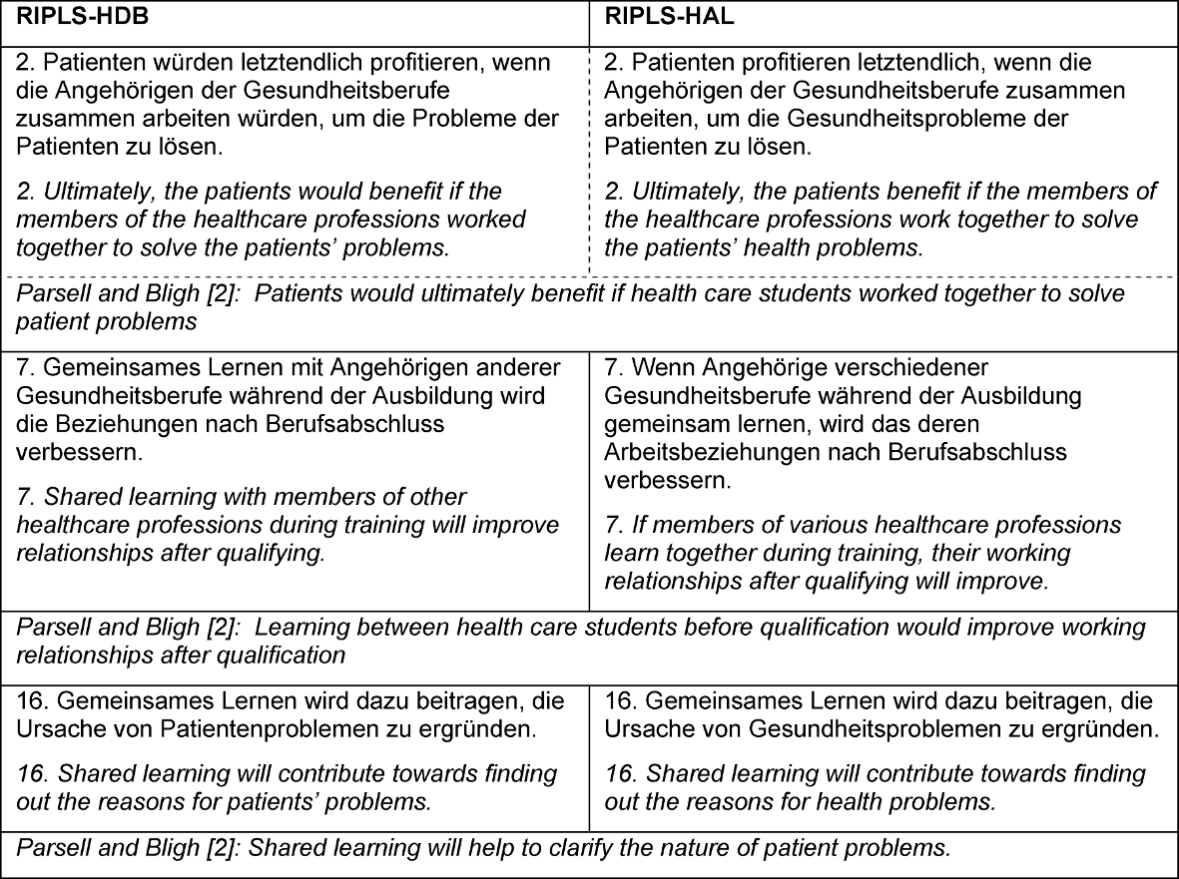
Comparison of the changed items in RIPLS-HDB and RIPLS-HAL

**Table 2:**
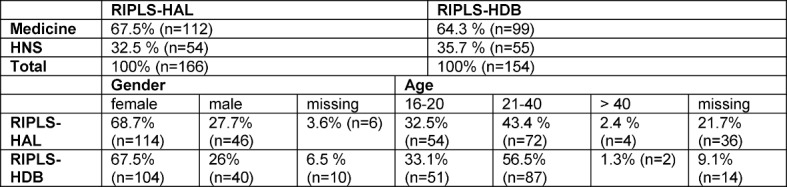
Descriptive characteristics of both samples

**Table 3:**
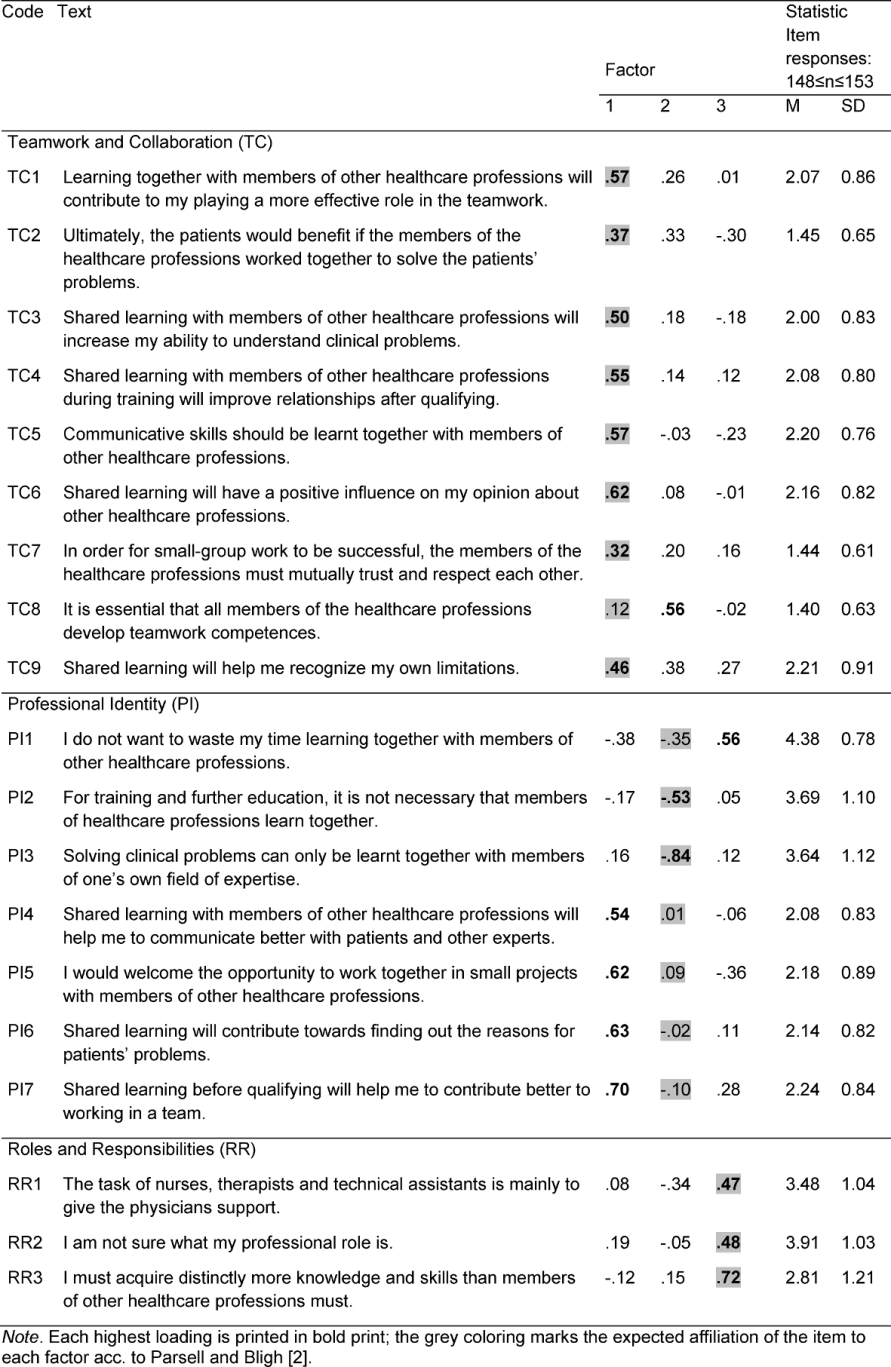
Factor loadings of the RIPLS-HDB acc. to Varimax-Rotation

**Table 4:**
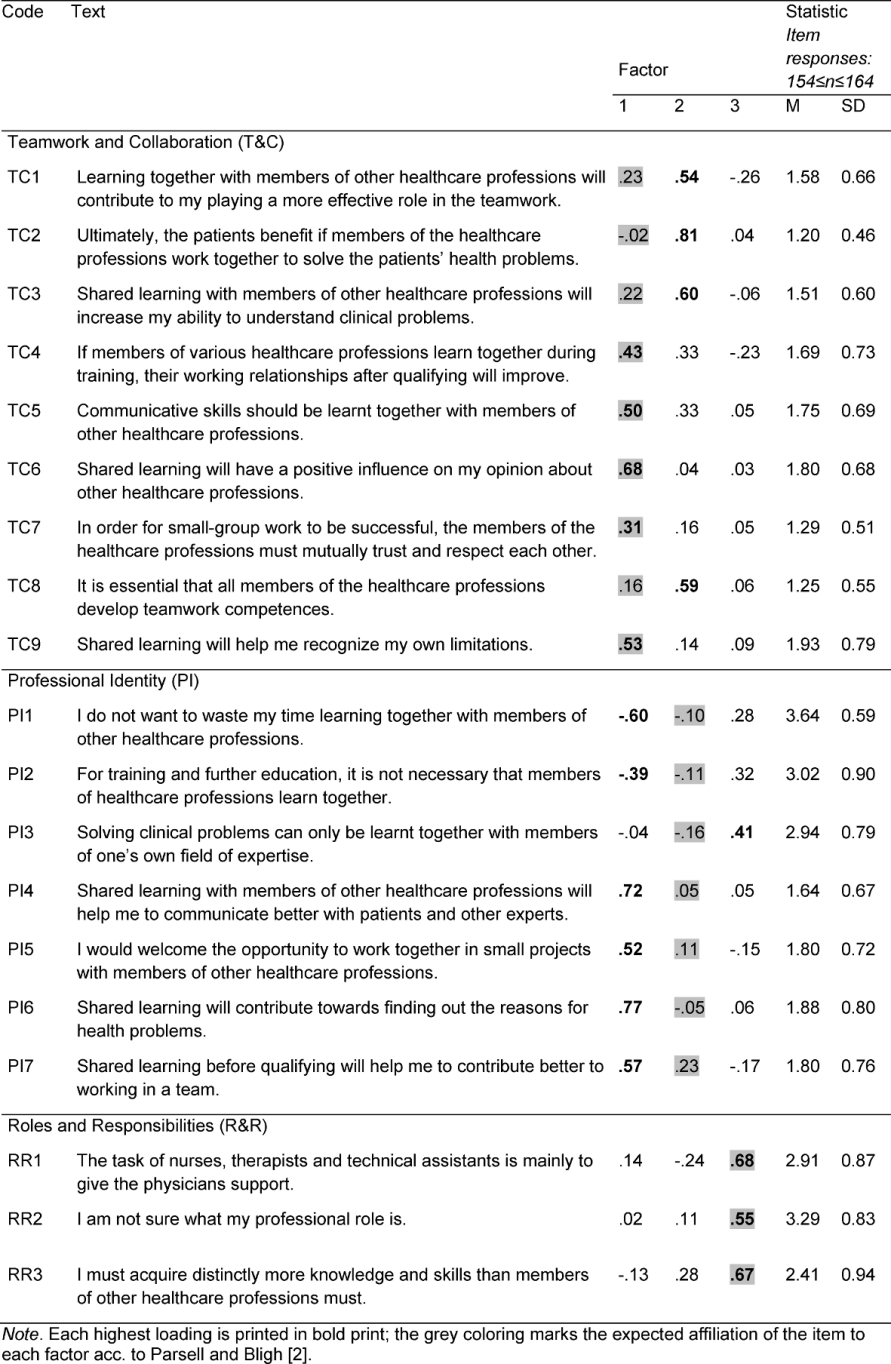
Factor loadings of the modified RIPLS Items (RIPLS-HAL) acc. to Varimax-Rotation
